# The role of perioperative warming in surgery: a systematic review

**DOI:** 10.1590/S1516-31802009000400009

**Published:** 2009-12-08

**Authors:** Muhammad Shafique Sajid, Ali Jabir Shakir, Kamran Khatri, Mirza Khurrum Baig

**Affiliations:** 1 MBBS, MBA, MD, MSc, FRCS. Specialist Registrar in General & Colorectal Surgery, Department of Colorectal Surgery, Worthing Hospital, Worthing, West Sussex, United Kingdom.; 2 MBBS, FRCS. Specialty Surgeon in General & Colorectal Surgery, Department of Colorectal Surgery, Worthing Hospital, Worthing, West Sussex, United Kingdom.; 3 MBBS, MRCS. Senior House Officer in General & Colorectal Surgery, Department of Colorectal Surgery, Worthing Hospital, Worthing, West Sussex, United Kingdom.; 4 MBBS, MD, FRCS. Consultant Surgeon in the Department of Colorectal Surgery, Worthing Hospital, Worthing, West Sussex, United Kingdom.

**Keywords:** Body temperature regulation, Hypothermia, Blood loss, surgical, Shivering, Wound infection, Regulação da temperatura corporal, Hipotermia, Perda sanguínea cirúrgica, Tremor por sensação de frio, Infecção dos ferimentos

## Abstract

**OBJECTIVE:**

The objective of this review was to systematically analyze the trials on the effectiveness of perioperative warming in surgical patients.

**METHODS:**

A systematic review of the literature was undertaken. Clinical trials on perioperative warming were selected according to specific criteria and analyzed to generate summative data expressed as standardized mean difference (SMD).

**RESULTS:**

Twenty-five studies encompassing 3,599 patients in various surgical disciplines were retrieved from the electronic databases. Nineteen randomized trials on 1785 patients qualified for this review. The no-warming group developed statistically significant hypothermia. In the fixed effect model, the warming group had significantly less pain and lower incidence of wound infection, compared with the no-warming group. In the random effect model, the warming group was also associated with lower risk of post-anesthetic shivering. Both in the random and the fixed effect models, the warming group was associated with significantly less blood loss. However, there was significant heterogeneity among the trials.

**CONCLUSION:**

Perioperative warming of surgical patients is effective in reducing postoperative wound pain, wound infection and shivering. Systemic warming of the surgical patient is also associated with less perioperative blood loss through preventing hypothermia-induced coagulopathy. Perioperative warming may be given routinely to all patients of various surgical disciplines in order to counteract the consequences of hypothermia.

## RESUMO

**OBJETIVO:**

O objetivo desta revisão é analisar sistematicamente os ensaios sobre a eficácia do aquecimento perioperatório em pacientes cirúrgicos.

**MÉTODOS:**

Uma revisão sistemática da literatura foi realizada. Ensaios clínicos sobre aquecimento perioperatório foram selecionados segundo critérios específicos e analisados para gerar dados sumativo expresso na diferença média padronizada (standardized mean difference, SMD).

**RESULTADOS:**

Vinte e cinco estudos englobando 3.599 pacientes de várias disciplinas de cirurgia foram obtidos a partir de bases de dados eletrônicas. Dezenove ensaios aleatórios em 1.785 pacientes qualificados para esta revisão. Nenhum grupo de aquecimento desenvolveu estatisticamente significativa hipotermia. No modelo de efeito fixo, grupo de aquecimento tiveram significativamente menos dor e menor incidência de infecção na ferida quando comparado com o grupo de não-aquecimento. No modelo de efeito aleatório, grupo de aquecimento também foi associado a um menor risco de tremores pós-anestesia. Em ambos os modelos de efeitos fixos e aleatórios, o aquecimento foi significativamente associado com menor perda de sangue. No entanto, houve significativa heterogeneidade entre os ensaios.

**CONCLUSÃO:**

O aquecimento perioperatório de pacientes cirúrgicos é eficaz na redução da dor pós-operatória ferida, infecção ferida, e tremores. O aquecimento sistêmico do paciente cirúrgico também está associado com menor perda de sangue no perioperatório prevenindo hipotermia e induzindo coagulopatia. O aquecimento perioperatório pode ser administrado rotineiramente a todos os pacientes cirúrgicos de diversas disciplinas, a fim de neutralizar as consequências da hipotermia.

## Introduction

Hypothermia, defined as core temperature below 36 °C^1^^,^^2^^,^^3^ is common in operating theaters and has often been disregarded as an inevitable consequence of general anesthesia and surgery.^2^^,^^4^^,^^5^ The body’s core temperature is determined by the balance between heat loss and heat gain. Exposure to a cold operating theater environment and anesthetic-induced impairment of thermoregulatory control are two of the commonest contributing factors that tip the balance in favor of heat loss, thereby leading to hypothermia in surgical patients.^1^^,^^6^

Hypothermia confers distinct benefits as well as severe complications in surgical patients. The potential benefits include protection against the deleterious effects of cerebral ischemia and malignant hyperthermia.^7^ However, hypothermia may increase susceptibility to perioperative wound infection by causing vasoconstriction and impaired immunity. Vasoconstriction decreases the partial pressure of oxygen in tissue, which lowers the resistance to infection.^8^ The other commonly known adverse effects of hypothermia include shivering,^9^ prolonged duration of drug action,^10^ coagulopathy,^11^ myocardial ischemia and decreased resistance to surgical infections.^12^ Perioperative warming has been shown to reduce perioperative complications.^13^^,^^14^ Several prophylactic and therapeutic measures have been tried with the aim of reducing or abolishing the development of perioperative hypothermia. Various perioperative warming techniques like simple cotton blankets, carbon-fiber sheets, circulating hot water mattresses, forced air warming, warm fluid infusion and esophageal heat exchange systems^9^^,^^15^^,^^16^ are in use in all surgical disciplines. These perioperative warming systems are being used during the preoperative, intraoperative and postoperative phases with variable efficacy. The duration of perioperative warming is also under review and prolonged exposure of surgical patients to warming systems has proven to be quite effective in major elective abdominal surgery.^17^

The aim of this systematic review was to compare the efficacy of perioperative warming of surgical patients aimed at reducing the consequences of wound infection, coagulopathy, blood loss, postoperative pain and postoperative shivering, in relation to no warming.

## Methods

Relevant prospective randomized controlled trials on perioperative warming among surgical patients published between January 1980 and June 2007 were identified through the Medical Literature Analysis and Retrieval System Online (Medline), Excerpta Medica (Embase), Cumulative Index to Nursing and Allied Health Literature (CINAHL), Cochrane library and Pubmed databases. The search strategy for target articles was not limited by time, age or gender. However, through frequent and thorough searching, it was noticed that there were no published comparative, non-randomized or randomized trials in the literature before 1980. The terms “randomized trials on perioperative warming”, “trials on perioperative warming” and “warming in surgical patients” were used in combination with the headings “surgical patients”, “forced air warming”, “thermoregulation in anesthetized patients” and “warming blankets”. Relevant articles referenced in these publications were obtained. The “related article” function was also used to widen the search criteria. All abstracts, comparative studies, randomized trials, non-randomized trials and citations that were firstly scanned through were reviewed comprehensively in accordance with the Quality of Reporting of Meta-analyses (QUORUM) template for the literature search. Each article was critically reviewed to assess its eligibility for inclusion or exclusion in this review.

Statistical analysis was performed by a senior statistician, using the Statistics for Windows software in Microsoft Excel 2007^®^. The methods used were Hedges G statistic for the calculation of standardized mean difference (SMD), the inverse variance method for the fixed effect model and the DerSimonian/Laired method for the random effect model. The estimate of the difference between the two techniques was pooled depending on the effect weights in the results, which were determined by the variance in each trial estimate. Forest plots were used for graphical displays of results from the meta-analysis: the square around the estimate represents the accuracy of the estimation (sample size) and the line represents the 95% confidence interval.

## Results

**Figure 1.**
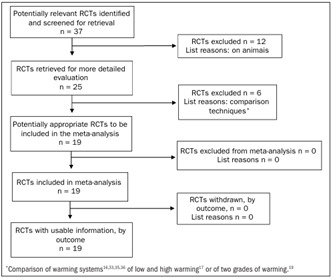
Quality of Reporting of Meta-Analyses (QUORUM) diagram template used in this review and results from the retrieval of randomized controlled trials (RCT).

Twenty-five studies encompassing 3,599 patients in various surgical disciplines were retrieved from the electronic databases. Nineteen randomized controlled trials^11^^,^^12^^,^^14^^,^^18^^,^^19^^,^^20^^,^^21^^,^^22^^,^^23^^,^^24^^,^^25^^,^^26^^,^^27^^,^^28^^,^^29^^,^^30^^,^^31^^,^^32^ on 1,785 patients qualified for this review in accordance with the inclusion criteria (Figure 1). Six trials^16^^,^^17^^,^^33^^,^^34^^,^^35^^,^^36^ were excluded for the reasons mentioned in Figure 1. The characteristics of the trials included are given in Table 1. ^11^^,^^12^^,^^14^^,^^18^^,^^19^^,^^20^^,^^21^^,^^22^^,^^23^^,^^24^^,^^25^^,^^26^^,^^27^^,^^28^^,^^29^^,^^30^^,^^31^^,^^32^

**Table 1.** Characteristics of included trials

Trial	Patients	Warming technique	Type of surgery	Outcome measurements
Melling and Leaper^18^	45	Non-contact radiant heat system	General surgery	Pain and wound infection
Kim et al.^19^	40	Forced warming blanket	Cardiothoracic	Temperature
Cavallini et al.^20^	76	Forced warming blanket	Plastic surgery	Temperature, coagulation
Zhao et al.^21^	40	Forced warming blanket	Abdominal surgery	Temperature, blood loss, shivering, extubation time
Scheck et al.^22^	30	Carbon-fiber warming blanket	Trauma patients	Temperature
Xu et al.^23^	40	Forced warming blanket and fluid warming device	Abdominal surgery	Temperature, blood loss, shivering, extubation time
Vanni et al.^24^	30	NA	Abdominal surgery	Temperature
Persson and Lundberg^25^	59	Forced warming blanket	Gynecological	Temperature, pain
Melling et al.^14^	421	Forced warming blanket	General surgery	Wound infection
El-Rahmany et al.^26^	149	Forced warming blanket	Cardiothoracic	Temperature, cardiovascular vital signs
Bock et al.^27^	40	Forced warming blanket	Abdominal Surgery	Temperature, blood loss, stay, cost, transfusion.
Wongprasartsuk et al.^28^	26	Forced warming blanket	Orthopedic	O_2_ consumption, CO_2_ production, pain, temperature
Frank et al.^12^	300	Forced warming blanket	Vascular, thoracic and abdominal	Temperature, ischemic heart disease, cardiac arrest
Schmied et al.^11^	60	Forced warming blanket	Orthopedics	Blood loss, transfusions
Kurz et al.^29^	200	Forced warming blanket	Colorectal surgery	Wound infection, stay
Frank et al.^30^	74	Forced warming blanket	Vascular, thoracic and abdominal	Neuroendocrine response, temperature, blood pressure, pulse rate
Frank et al.^31^	100	NA	Vascular surgery	Temperature, cardiac stress
Camus et al.^32^^*^	22	Forced warming blanket	Abdominal Surgery	Temperature, shivering
Camus et al.^32^^†^	33	Forced warming blanket	Abdominal surgery	Temperature, shivering

*limb a of trial; †limb b of trial. NA = not available. Heating technique was not mentioned in the trial but this group was definitely provided with perioperative warming.

### Methodological quality of studies included

**Chart 1.**
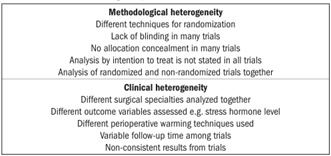
Causes of heterogeneity

The characteristics of the trials included are explained comprehensively in Table 2 for methodological quality analysis. ^11^^,^^12^^,^^14^^,^^18^^,^^19^^,^^20^^,^^21^^,^^22^^,^^23^^,^^24^^,^^25^^,^^26^^,^^27^^,^^28^^,^^29^^,^^30^^,^^31^^,^^32^ The Mantel-Haenszel fixed effect model was used to compute robustness and susceptibility to any outlier among these trials. The allocation, concealment and blinding of the investigator or assessor were not clearly reported, and consequently the methodological quality of the trials was considered inadequate and the results from our review may be considered biased. Heterogeneity (clinical and methodological diversity) was seen among all these trials (Chart 1). Limited availability of data on various outcome variables and lack of a major multicenter double blind randomized controlled trial restricted this review with regard to detailed sub-group analysis. However, a subgroup analysis of trials with clearly reported allocation concealment was performed. We felt that performing sensitivity analysis was not relevant due to limited numbers of studies. We attempted to assess publication bias by using funnel plots, but this was difficult to compute due to the small numbers of patients.

**Table 2.** The randomized controlled trials included, all of them with stated inclusion and exclusion criteria

Trial	Baseline comparables	Blinding	Technique of randomization	Allocation concealment	Intention to treat analysis
Melling and Leaper^18^	Stated	Yes	Random number technique	Yes	No
Kim et al.^19^	Stated	No	Sealed envelopes	Yes	No
Cavallini et al.^20^	Stated	No	Random assigning	No	No
Zhao et al.^21^	Stated	No	Not given	No	No
Scheck et al.^22^	Stated	No	Not given	No	No
Xu et al.^23^	Stated	No	Not given	No	No
Vanni et al.^24^	Stated	Yes	Sealed envelopes	Yes	No
Melling et al.^14^	Stated	No	Sealed envelopes	No	Yes
Persson and Lundberg^25^	Stated	No	Not given	No	No
El-Rahmany et al.^26^	Stated	No	Computerized	No	Yes
Bock et al.^27^	Stated	No	Random assigning	No	Yes
Wongprasartsuk et al.^28^	Stated	No	Random assigning	Yes	Yes
Frank et al.^12^	Stated	No	Computerized	No	No
Schmied et al.^11^	Stated	No	Random assigning	Yes	Yes
Kurz et al.^29^	Stated	Yes	Not given	No	No
Frank et al.^30^	Stated	Yes	Computerized	No	No
Frank et al.^31^	Stated	No	Computerized	No	No
Camus et al.^32^	Not stated	No	Not given	No	No

RCT = randomized controlled trial.

### Hypothermia

**Figure 2.**
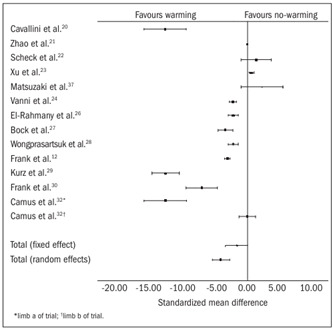
Hypothermia: combined analysis of the randomized controlled trials in the review.

Fourteen trials^12^^,^^20^^,^^21^^,^^22^^,^^23^^,^^24^^,^^26^^,^^27^^,^^28^^,^^29^^,^^30^^,^^31^^,^^32^^,^^37^ contributed towards the combined analysis on the development of hypothermia in the no-warming group. In both the fixed and the random effect models, the no-warming group developed statistically significant hypothermia [fixed effect SMD -1.78, 95% confidence interval, CI (-1.96, -1.61), P = 0.0000, degrees of freedom, df = 13, z = -20.25; and random effect SMD -4.44, 95% CI (-5.92, -2.95), P = 0.0000, df = 13, z = -5.92; Table 3 ^12^^,^^20^^,^^21^^,^^22^^,^^23^^,^^24^^,^^26^^,^^27^^,^^28^^,^^29^^,^^30^^,^^32^^,^^37^ and Figure 2].

**Table 3.** Temperature changes: combined analysis

	Warming group	Control group
Cavallini et al.^20^	36 ± 0.6 C	34 ± 1.0
Zhao et al.^21^	36.4 ± 0.4 C	35.3 ± 0.5 C
Scheck et al.^22^	36.4 ± 0.2 C	34.7 ± 0.6 C
Xu et al.^23^	36.4 ± 0.4 C	35.3 ± 0.5 C
Vanni et al.^24^	34.2 ± 1.1 C	34.1 ± 0.9 C
El-Rahmany et al.^26^	34.5 ± 0.1 C	34.5 ± 0.1 C
Bock et al.^27^	36.5 C	35.5 C
Wongprasartsuk et al.^28^	36.9 ± 0.55 C	36.2 ± 0.87 C
Frank et al.^12^	36.7 ± 0.1 C	35.4 ± 0.1 C
Kurz et al.^29^	36.6 ± 0.5 C	34.7 ± 0.6 C
Frank et al.^30^	36.7 ± 0.1 C	35.3 ± 0.1 C
Camus et al.^32^^*^	36.4 ± 0.1 C	34.6 ± 0.3 C
Camus et al.^32^^†^	37.1 ± 0.1 C	35.1 ± 0.2 C
Matsuzaki et al.^37^	36.9 ± 0.3 C	36.6 ± 0.5 C

*limb a of trial; †limb b of trial.

### Postoperative pain

**Figure 3.**
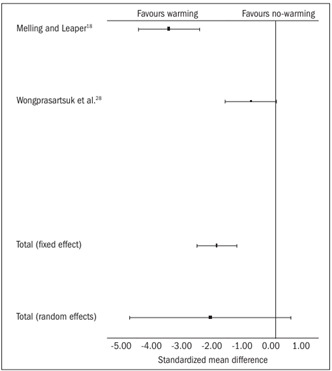
Postoperative pain: combined analysis of the randomized controlled trials in this review.

Two trials^18^^,^^28^ contributed towards the combined analysis on postoperative pain. In the fixed effect model, the warming group had significantly less pain [SMD -1.84, 95% CI (-2.45, -1.22), P = 0.0000, df = 1, z = -5.8]. In the random effect model, this difference was not statistically significant between the two groups [SMD -2.0, 95% CI (-4.5, 0.46), P = 0.11, df = 1, z = -1.59; Table 4 ^18^^,^^28^ and Figure 3]. However, there was significant heterogeneity among the trials (Q = 16.28, P = 0.001).

**Table 4.** Postoperative pain: combined analysis

Mean pain score (Visual Analogue Scale) 0-10 cm		
	Warming group	Control group
Melling and Leaper^18^	2.8	4.5
Wongprasartsuk et al.^28^	5.7	6.1

### Wound infection

**Figure 4.**
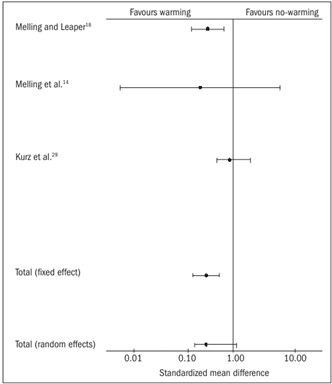
Wound infection: combined analysis of the randomized controlled trials in this review.

Three trials^14^^,^^18^^,^^29^ contributed towards the combined analysis on the postoperative wound infection rate. In the fixed effect model, the warming group was associated with lower risk of developing postoperative wound infection [SMD 0.32, 95% CI (0.18-0.56), P = 0.0001, df = 2, z = -3.99; Table 5 ^14^^,^^18^^,^^29^ and Figure 4], compared with the no-warming group. There was no heterogeneity among the trials (Q = 0.06, P = 0.96).

**Table 5.** Wound infection: combined analysis

Wound infection frequency		
	Warming group	Control group
Melling et al.^14^	13/277	19/139
Melling and Leaper^18^	0/30	1/15
Kurz et al.^29^	6/104	18/96

### Shivering

**Figure 5.**
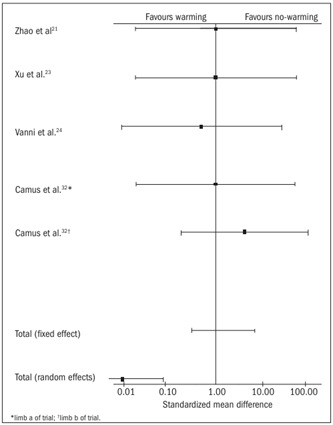
Shivering: combined analysis of the randomized controlled trials in this review.

Five trials^21^^,^^23^^,^^24^^,^^32^ contributed towards the combined analysis on postoperative shivering. In the random effect model, the warming group was associated with lower risk of post-anesthetic shivering [SMD 0.01, 95% CI (0.001-0.08), P = 0.0000, df = 4, z = -4.43; Table 6 ^21^^,^^23^^,^^24^^,^^32^ and Figure 5], compared with the no-warming group. There was no heterogeneity among the trials (Q = 0.082, P = 0.9980).

**Table 6.** Trials on postoperative shivering: combined analysis

	Warming group	Control group
Zhao et al.^21^	0/20	6/20
Xu et al.^23^	0/20	6/20
Vanni et al.^24^	0/20	5/10
Camus et al.^32^^*^	1/11	9/11
Camus et al.^32^^†^	2/22	7/22

*limb a of trial; †limb b of trial.

### Blood loss

**Figure 6.**
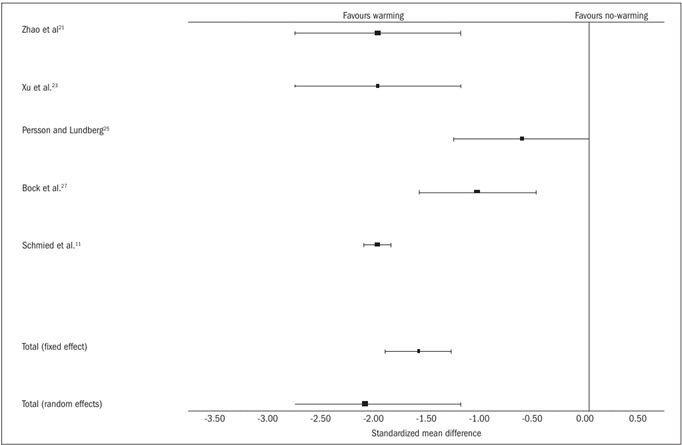
Blood loss: combined analysis of the randomized controlled trials in this review.

Five trials^11^^,^^21^^,^^23^^,^^25^^,^^27^ contributed towards the combined analysis on perioperative blood loss. Both in the random and in the fixed effect models, the warming group was associated with significantly less blood loss [random effect SMD -1.60, 95% CI (-1.92, -1.29), P = 0.0000, df = 4, z = -9.99; and fixed effect SMD -2.10, 95% CI (-3.31, -0.89), P = 0.0007, df = 4, z = -3.40; Table 7 ^11^^,^^21^^,^^23^^,^^25^^,^^27^ and Figure 6]. However, there was significant heterogeneity among the trials (Q = 55.77, P = 0.0000).

**Table 7.** Trials on blood loss: combined analysis

	Warming group	Control group
Zhao et al.^21^	112 ± 80 ml	350 ± 145 ml
Xu et al.^23^	112 ± 80 ml	350 ± 145 ml
Persson and Lundberg^25^	108 ± 27 ml	308 ± 47 ml
Bock et al.^27^	635 ± 507 ml	1070 ± 803 ml
Schmied et al.^11^	1670 ± 320 ml	2150 ± 550 ml

### Myocardial dysfunction, coagulopathy and stress hormone imbalance

There was insufficient data in the trials available to assess hypothermia-induced myocardial dysfunction, coagulopathy and stress hormone imbalance.

### Subgroup analysis

In the subgroup analysis, trials with allocation concealment^2^^,^^11^^,^^19^^,^^24^^,^^28^ were analyzed separately. Two trials^24^^,^^28^ contributed towards the calculation of hypothermia. The warming group was at less risk of developing hypothermia, compared with the no-warming group (P = 0.0163). Two trials^18^^,^^28^ contributed towards the calculation of postoperative pain. The warming group was associated with less postoperative wound pain, compared with the non-warming group (P = 0.0001). The combined calculation of perioperative blood loss, wound infection and postoperative shivering could not be performed because of insufficient data quoted in the trials.

## Discussion

Patients in various surgical disciplines are exposed to numerous factors that may alter thermoregulatory mechanisms and result in postoperative hypothermia, including a cold operating theater, cold intravenous fluids, cold blood transfusions, cold antiseptic skin preparations and anesthesia.^1^^,^^38^^,^^39^ The latter obliterates behavioral responses and inhibits afferent input, thereby lowering the temperature threshold for thermoregulatory responses to hypothermia and preventing efferent responses.^40^ Some patients are particularly at higher risk of developing hypothermia: the factors involved include surgery lasting for more than two hours, extremes of age, trauma, abdominal surgery, thoracic surgery, massive transfusions of intravenous fluids or blood and massive blood or fluid loss.^38^^,^^39^ Inadvertent perioperative hypothermia prolongs the recovery time and also increases blood loss, surgical site infection and total hospital stay.^8^^,^^39^

Perioperative skin warming has been shown to reduce the initial postinduction hypothermia, intraoperative hypothermia and postoperative shivering, even for procedures lasting for more than three hours.^9^ Furthermore, a single hour of preoperative skin surface warming has been reported to reduce the rate at which core hypothermia developed during the first hour of anesthesia.^33^ Our analysis shows that the no-warming group is at significant risk of developing perioperative hypothermia, which in turn can give rise to significant perioperative morbidity.

Perioperative systemic warming, in addition to standard forced warm air intraoperative warming, significantly reduces blood loss and complications in patients.^17^ These findings corroborate those from the independent studies of Schmied et al.^11^ and Winkler et al.^34^ In the latter study on blood loss following total hip arthroplasty, even a small difference in median core intraoperative temperature of 0.5 °C resulted in significantly less blood loss among the patients who were warmed. This excessive blood loss in hypothermic patients is due to hypothermia-induced coagulopathy^41^^,^^42^ that results from impaired platelet aggregation and prolonged bleeding time. Bleeding time depends on several variables, including the number and function of platelets, white and red cell counts, vascular factors, hormones and temperature. Although studies have been widely conducted, the bleeding time test does not strictly correlate with surgical bleeding.^41^^,^^43^ Nonetheless, with standardized techniques and knowledge of the merits and limitations of the bleeding time test, it is useful for diagnosing hemostasis disorders, guiding their therapy and warning of unexpected bleeding complications in surgical patients.^44^ Stensrud et al.^45^ evaluated the effects of intraoperative hypothermia on blood transfusion during cardiac surgery. They reported that even though no differences in total blood requirements were reported between patients receiving a normothermic cardiopulmonary bypass and those receiving a hypothermic bypass, the hypothermic patients showed an activated partial thromboplastin time that was prolonged by nearly 8%, compared with patients who were actively warmed. No differences were observed in prothrombin time and fibrinogen concentrations. Our study confirms that perioperative warming can significantly reduce bleeding following surgery and that it may be recommended for regular use.

The risk of wound infection in patients undergoing colonic surgery ranges from 9-27%^46^ and it may be reduced by two-thirds among patients who receive perioperative warming.^8^^,^^46^ By extending the warming period, to two hours before and after surgery, the incidence of wound infection can be further reduced from 27% to 13% and overall complications can be reduced from 54% to 32%.^17^ Our review concludes that perioperative warming can significantly reduce the incidence of wound infection.

There was significant heterogeneity among the trials (Chart 1). There may be many reasons for heterogeneity, including combined analysis on trials from various surgical disciplines, combined analysis on trials in which different types of anesthesia (general, spinal or combined epidural and spinal) are used in variable doses and inclusion of trials in which warming was given to different parts of the body. The results from the studies included in this review were also inconsistent. No major multicenter, randomized, controlled trial was reported in the literature. Thus, it was difficult to find high quality, unbiased data for analysis. Nonetheless, this is the only reported systematic review on the role of perioperative warming among surgical patients.

## Conclusion

In conclusion, perioperative warming of surgical patients is effective for reducing postoperative wound pain, wound infection and shivering. Systemic warming of surgical patients is also associated with less perioperative blood loss, by preventing hypothermia-induced coagulopathy. Perioperative warming may be given routinely to all patients in various surgical disciplines in order to counteract the consequences of hypothermia.
